# A case report of adenomyosis-induced spontaneous rupture in an unscarred and unpregnant uterus

**DOI:** 10.1097/MD.0000000000041037

**Published:** 2024-12-20

**Authors:** Yan Liu, Xiaoling Hu, Wen Lv, Yingzi Xu

**Affiliations:** a Department of Ultrasound, Tongde Hospital of Zhejiang Province, Hangzhou, China; b Department of Gynecology, Tongde Hospital of Zhejiang Province, Hangzhou, China.

**Keywords:** adenomyosis, ultrasound examination, uterine rupture

## Abstract

**Rationale:**

Spontaneous uterine rupture, although rare, is a life-threatening obstetric emergency with a high maternal and fetal mortality rate. It can occur without warning, leading to severe complications, including hemorrhage, shock, and fetal demise. The risk factors contributing to uterine rupture are diverse and include a history of uterine surgery (such as cesarean section), trauma to the uterus, abnormal uterine contractions during labor, and underlying conditions like adenomyosis. Identifying and understanding these risk factors are crucial for early detection, timely intervention, and improved outcomes in affected pregnancies.

**Patient concerns:**

Here, we report an exceptionally rare case of spontaneous uterine rupture triggered by adenomyosis in a 34-year-old unmarried and nulliparous woman with no prior scarring or pregnancy. Physical examination revealed abdominal distension, with the uterine fundus at the level of the umbilicus, exhibiting hardness, tenderness upon palpation, and rebound tenderness. Ultrasonography and computerized tomography scans suggested adenomyosis and uterine rupture. During surgery, a rupture was discovered in the left basal layer of the uterus, with a rupture diameter of approximately 3 cm. Additionally, there were multiple internal endometrial lesions in the uterorectal pouch.

**Diagnoses:**

Adenomyosis-induced spontaneous uterine rupture.

**Interventions:**

The procedures performed included excision of adenomyotic lesions, repair of the uterine rupture, and electrocoagulation of endometriotic lesions in the pelvic cavity.

**Outcomes:**

At the 1-month post-surgery follow-up examination, the patient showed good recovery, with no signs of complications. She was able to resume normal daily activities without difficulty. The surgical site was healing well, with no signs of infection or abnormal scarring. Ultrasound and clinical assessments confirmed the resolution of pelvic fluid accumulation, and uterine function appeared to be intact. The patient was advised to continue regular follow-up visits to monitor her recovery and ensure long-term well-being.

**Lessons:**

First, uterine rupture caused by adenomyosis in a nonpregnant, scarless uterus is extremely rare but still possible. When patients with severe adenomyosis present with abdominal pain and pelvic fluid, obstetricians and gynecologists should consider the possibility of uterine rupture. Second, ultrasound examination can quickly and accurately diagnose both adenomyosis and uterine rupture.

## 1. Introduction

Adenomyosis is a prevalent benign gynecological disease characterized by the presence of endometrial glands and stroma deep within the myometrium. This condition often manifests with symptoms such as dysmenorrhea, infertility, and dyspareunia.^[1,2]^ Previous research^[3–6]^ suggest that adenomyosis may be associated with miscarriage, preterm birth, fetal growth restriction, and poor fetal outcomes in pregnant women. Additionally, there are reports^[7–9]^ indicating that adenomyosis in pregnant women can lead to uterine rupture. However, it is crucial to note that there have been no reported cases of spontaneous rupture in an unscarred, nonpregnant uterus caused by adenomyosis, underscoring the rarity and significance of our case.

## 2. Case

**Figure 1.**
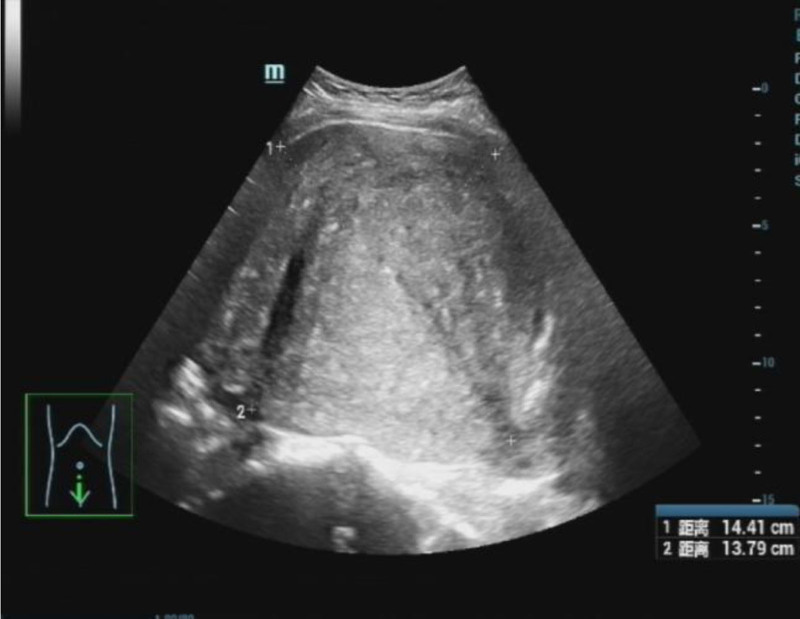
The ultrasound revealed a uterus displaying signs of adenomyosis.

**Figure 2.**
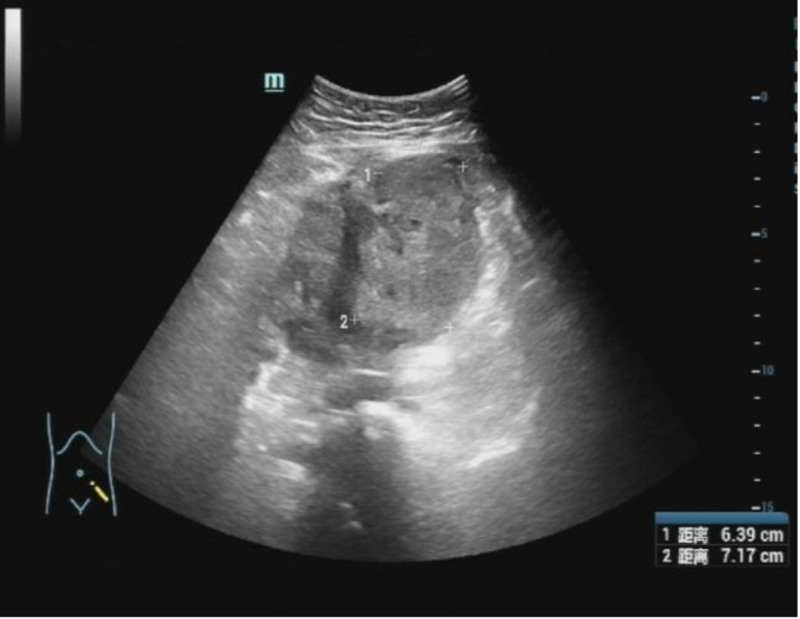
Huge hematoma was observed in the ultrasonography.

**Figure 3.**
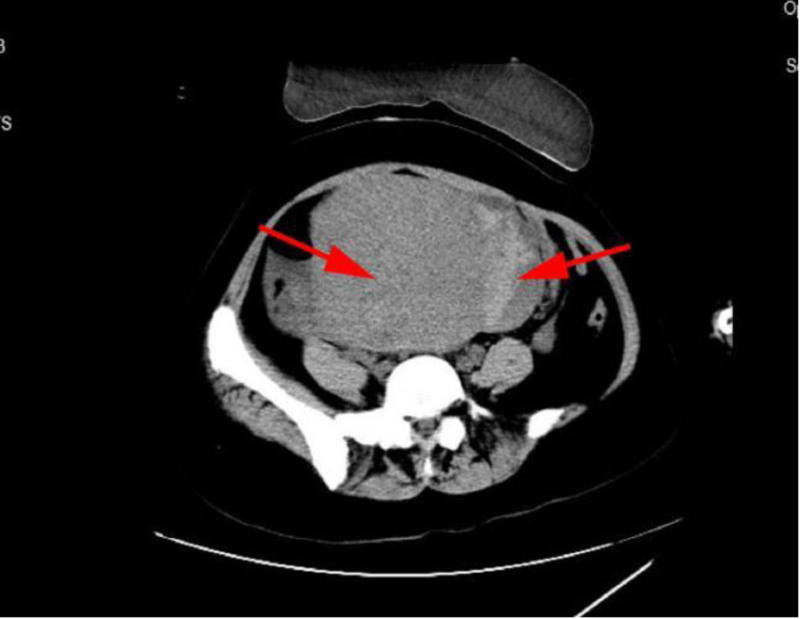
The CT scan revealed adenomyosis in the lower part of the uterus (left arrow), along with hematoma adjacent to it (right arrow). CT = computerized tomography.

In March 2024, a 34-year-old nulliparous woman with a 9-year history of adenomyosis and anemia presented to the hospital with acute abdominal pain during menstruation. She had been undergoing long-term hormone therapy. Even though she claimed no previous hypertension or diabetes, she came to our obstetrics and gynecology emergency department for medical attention. Preadmission ultrasound revealed a uterus measuring approximately 14 × 13 × 12 cm, with diffuse thickening and uneven distribution of the myometrial layer (Fig. 1). An irregular mixed echoic mass, approximately 6 × 10 × 7 cm in size, was observed adjacent to the uterine base (Fig. 2), showing no blood flow signal on color Doppler imaging. The patient also exhibited a small amount of intraperitoneal fluid. These findings suggested adenomyosis with possible uterine rupture. Computerized tomography (CT) imaging confirmed a significantly enlarged uterus (~6.6 cm in diameter) with adjacent low-density shadows, suggesting uterine rupture (Fig. 3). Laboratory results upon admission showed a hemoglobin level of 64 g/L, white blood cell count of 13.7 10E9/L, platelet count of 243 10E9/L, temperature of 37.4 °C, pulse rate of 102 beats per minute, respiratory rate of 20 breaths per minute, and blood pressure of 150/90 mm Hg. Physical examination revealed abdominal distension, with the uterine fundus at the level of the umbilicus, exhibiting hardness, tenderness upon palpation, and rebound tenderness.

**Figure 4.**
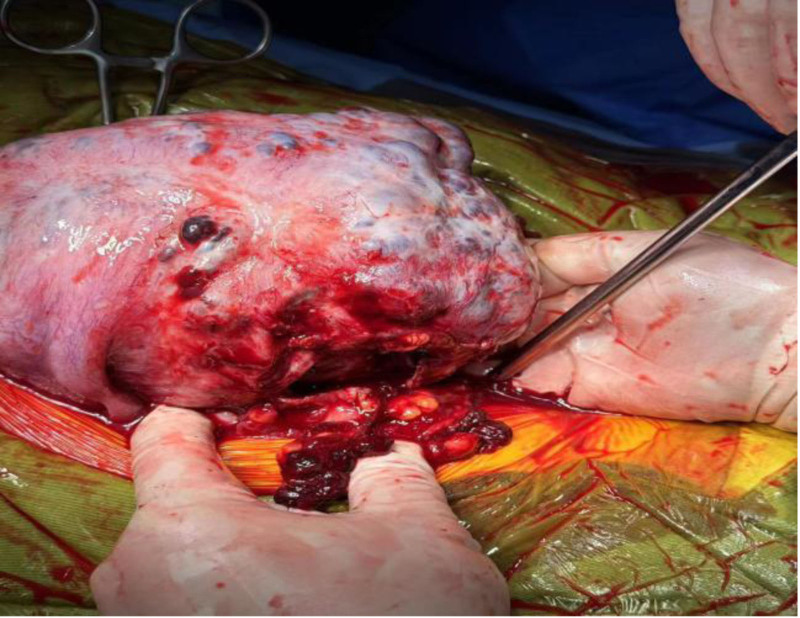
The intraoperative macro image shows a rupture of the left floor of the uterus.

As a result, the patient was expeditiously transferred to the operating room. During surgery, a notably enlarged uterus resembling a gestational age of around 5 months was observed. Multiple purplish-blue cystic lesions were noted protruding from the uterine surface, with the largest measuring approximately 3 cm in diameter. The uterine surface exhibited abundant, engorged blood vessels. Adhesions were noted between the posterior uterine wall and the omentum and intestines, with a 3 cm diameter active bleeding rupture identified upon separation of the adhesions (Fig. 4). Both fallopian tubes appeared normal, and no abnormalities were detected in the ovaries. Scattered endometrial ectopic lesions were visible in the uterorectal recess. In the pelvic and abdominal cavities, a hematoma approximately 10 × 10 cm surrounding the root of the sigmoid colon mesentery was observed.

**Figure 5.**
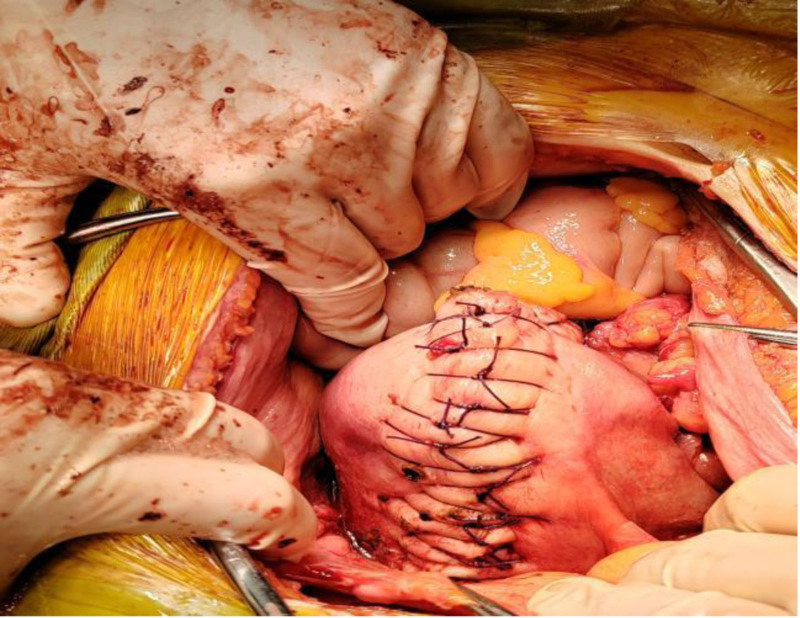
The intraoperative macro image depicted the adenomyosis lesion after it was removed during uterine repair.

**Figure 6.**
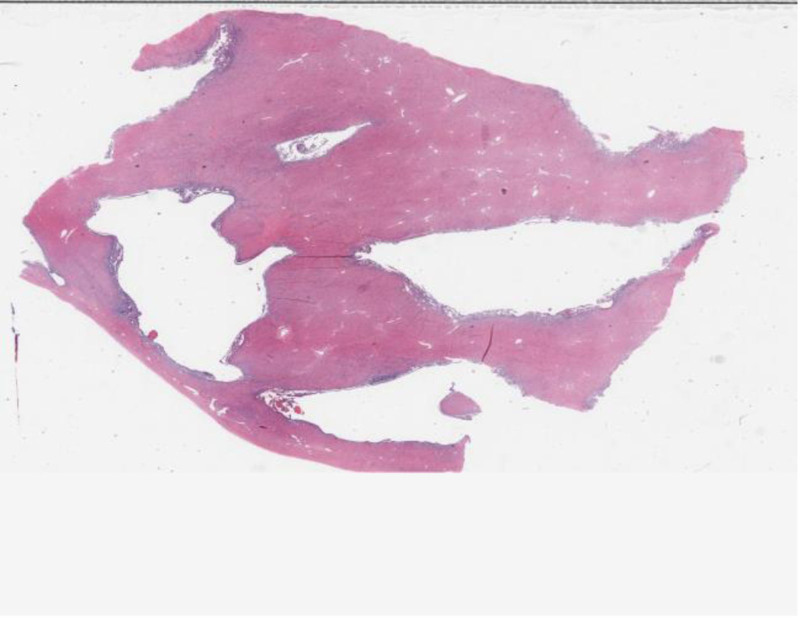
High-power tissue microscopy revealed inner membrane glands within the muscle fibers (hematoxylin–eosin stain, original magnification: 10×).

Considering the patient’s unmarried and nulliparous status, along with her desire for future fertility, the surgical approach comprised excision of adenomyotic lesions, repair of the uterine rupture, electrocoagulation of endometriotic lesions in the pelvic cavity (Fig. 5). Pathological examination confirmed widespread diffuse adenomyosis extending through the uterine wall, particularly involving the posterior uterine wall and uterine base, consistent with the site of uterine rupture (Fig. 6). The patient has now fully recovered and has been discharged from the hospital.

## 3. Discussion

The spontaneous rupture of an unscarred uterus is a rare gynecological and obstetric emergency, mainly occurring in the mid to late stages of pregnancy.^[10–12]^ There have been no reported cases of spontaneous rupture in an unscarred, nonpregnant uterus caused by adenomyosis. In our case, the patient also presented with endometriotic lesions in the uterorectal pouch, making it exceptionally rare. The exact cause of uterine rupture induced by adenomyosis remains unclear. However, it is hypothesized that ectopic endometrium within the myometrium induces hypertrophy and thickening of the surrounding muscle layers, leading to diffuse enlargement of the uterus.^[13]^ This results in damaged, thickened, and less elastic muscle layers.^[14]^ Additionally, adenomyosis secretes inflammatory substances, further compromising the uterine muscle layers.^[13]^ Penetration of adenomyotic lesions through the uterine muscle layers can cause uterine rupture and bleeding, which can lead to hemorrhagic shock, endangering the patient’s life. Therefore, uterine rupture is a critical gynecological and obstetric emergency. Therefore, early and accurate diagnosis is very important.

Currently, the main auxiliary examinations for adenomyosis in clinical practice include ultrasound, CT, and magnetic resonance imaging. Compared to CT and magnetic resonance imaging, ultrasound is more real-time, convenient, rapid, and cost-effective, facilitating dynamic continuous observation and bedside operations. Particularly for adenomyosis patients who have not yet exhibited obvious clinical symptoms, ultrasound diagnosis is more prominent, becoming the preferred examination method for adenomyosis.^[15]^

Typical ultrasound manifestations of adenomyosis include thickening and hypertrophy of the uterine muscle layers, some of which may appear cystic and show trabecular changes, with unclear lesion boundaries and abundant blood flow signals. Lesions can be diffuse or focal, with common locations being the posterior uterine wall and uterine base, which are also common sites of uterine rupture. Ultrasound examination can provide 7 aspects of information describing adenomyotic lesions, including the presence of adenomyosis, its location, the extent of lesions, cystic or non-cystic changes, depth of invasion into the muscle layer, and blood flow within the lesion.^[9,16–18]^ The information provided by ultrasound is essential for obstetricians and gynecologists to fully understand the condition and play a crucial role in personalized treatment for patients.^[19,20]^

At the same time, our study has some limitations. Our case is based on individual case analysis, and due to its rarity, we are unable to calculate the incidence rate of uterine rupture caused by adenomyosis. As a result, obstetricians and gynecologists cannot provide specific quantitative indicators when offering patient counseling.

## 4. Conclusions

Adenomyosis, defined by the presence of endometrial glands and stroma within the uterine musculature, warrants careful evaluation by gynecologists. However, uterine rupture caused by adenomyosis is extremely rare. Serious adenomyosis in patients with acute abdominal pain and pelvic fluid should be made aware of its potential association with uterine rupture, serving as a crucial risk factor. It is noteworthy that even in cases involving an unscarred and nonpregnant uterus, the risk of rupture persists. Obstetricians and gynecologists must be aware of this fact in order to provide more comprehensive and personalized diagnostic and treatment advice to patients. Adenomyosis and uterine rupture can be diagnosed quickly and accurately by ultrasound examination. Gynecologists can give patients timely diagnosis and reasonable treatment according to the results of ultrasound examination, and thus, reducing the mortality rate associated with uterine rupture.

## Acknowledgments

The authors extend their sincere appreciation to the patient for her participation, which made this work possible, and to the professionals and researchers involved in the study. Informed consent was obtained from the patient.

## Author contributions

**Conceptualization:** Yingzi Xu.

**Investigation:** Xiaoling Hu.

**Resources:** Wen Lv.

**Writing – original draft:** Yan Liu.

**Writing – review & editing:** Yingzi Xu.
